# Gut Microbiota and Metabolites: Biomarkers and Therapeutic Targets for Diabetes Mellitus and Its Complications

**DOI:** 10.3390/nu17162603

**Published:** 2025-08-11

**Authors:** Kai Yan, Xin Sun, Xin Wang, Jing Zheng, Hongsong Yu

**Affiliations:** 1 Special Key Laboratory of Ocular Diseases of Guizhou Province, Department of Immunology, Zunyi Medical University, Zunyi 563000, China; yankai0909@163.com (K.Y.); zhenjing@zmu.edu.cn (J.Z.); 2 Special Key Laboratory of Gene Detection and Therapy of Guizhou Province, School of Basic Medical Sciences, Zunyi Medical University, Zunyi 563000, China; sunxin@zmu.edu.cn (X.S.); wangxin@zmu.edu.cn (X.W.)

**Keywords:** diabetes mellitus, gut microbiota, diabetic complications, traditional Chinese medicine, diets

## Abstract

Diabetes mellitus (DM) is a complex metabolic disease characterized by significantly elevated blood glucose levels as a result of dysfunctional or impaired pancreatic β-cells, leading to insulin deficiency. This condition can result in severe complications, including cardiovascular diseases, kidney failure, vision impairment, and nerve damage. Currently available anti-diabetic drugs do not fully prevent the progression of these complications. Moreover, they often have significant side effects. The gut microbiota plays a crucial role in influencing diet, energy metabolism, and blood glucose levels. Research shows a strong link between microbiota dysbiosis and DM, as well as the severity of its complications. Commensal bacteria can help manage blood glucose levels, reduce inflammation, regulate metabolism, and enhance the gut barrier. Conversely, opportunistic pathogens can worsen insulin resistance, promote metabolic disorders, disrupt gut integrity, and affect appetite and weight. This article describes the characteristics of gut microbiota in various types of DM and explores the role of the “gut microbiota–metabolite–signaling pathway” axis in DM and its complications. In addition, it highlights the therapeutic potential of traditional Chinese medicine and dietary interventions through modulation of the gut microbiota and metabolites. The aim is to provide comprehensive evidence supporting the integration of TCM dietary therapy, targeted dietary strategies, and specific probiotics as alternative and complementary therapies for DM and its complications.

## 1. Introduction

Studies show that between 1990 and 2019, the global incidence rate of diabetes mellitus (DM) was on the rise year by year [1]. The incidence of type 2 diabetes mellitus (T2DM) in adolescents nearly tripled compared to pre-pandemic levels during COVID-19 [2], and the adult T2DM incidence rate also shows the same trend [3]. This may be related to virus-induced pancreatic beta-cell damage through the ACE2 receptor [4]. DM mainly includes type 1 diabetes mellitus (T1DM), T2DM, and gestational diabetes mellitus (GDM). T1DM is primarily caused by the immune system mistakenly attacking pancreatic beta cells (β-cells), leading to insufficient insulin secretion. This process may be triggered by factors such as viral infections, dietary components, and chemical toxins. T2DM is characterized by insulin resistance (IR), and during pregnancy, hormones secreted by the placenta, such as placental lactogen, growth hormone, estrogen, and progesterone, increase IR. When the body’s insulin cannot lower blood glucose to the required level, it will lead to the occurrence of GDM [5]. Continuous elevation of blood glucose levels in the body leads to protein glycation and tissue damage, resulting in various complications. Diabetic retinopathy (DR) and diabetic kidney disease (DKD) are mainly caused by long-term hyperglycemia leading to microvascular damage. Diabetes neuropathy (DN) is a neuropathy caused by hyperglycemia, while diabetic cardiovascular disease (DCD) is the result of hyperglycemia, hypertension, and hyperlipidemia [6]. The management of DM primarily involves pharmacological treatment, lifestyle changes, and nutritional interventions [7]. Pharmacological treatment includes oral hypoglycemic agents and insulin injections, which help control blood glucose by reducing hepatic glucose production and increasing insulin sensitivity [8]. Lifestyle changes focus on maintaining a healthy weight and engaging in moderate-intensity physical activities such as walking, cycling, and swimming. Nutritional intervention emphasizes a balanced diet such as a low-sugar, low-fat, and high-fiber diet (HFD), as well as fat-soluble active components like prebiotics, probiotics, and coenzyme Q10, enabling patients to self-manage blood glucose levels [9].

**Figure 1:**
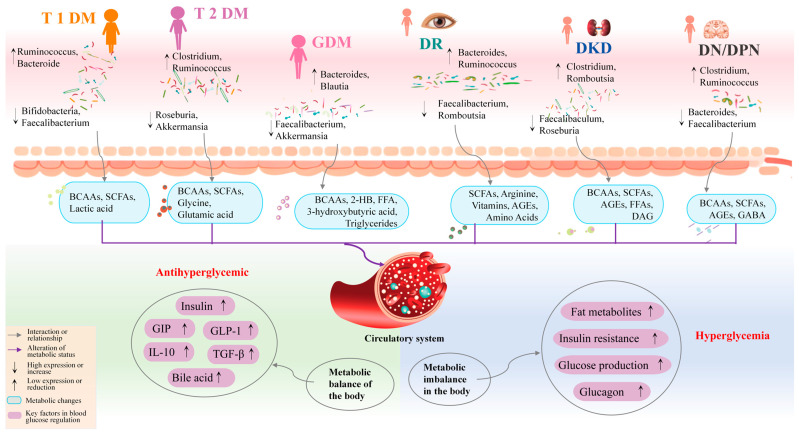
The gut microbiota in different types of DM regulates blood glucose levels by modulating the expression of metabolites. The differences in microbial community abundance, as shown in the figure, may be key factors for distinguishing between types of DM. These microbial communities influence the host’s metabolic profile, affecting glucose and lipid metabolism, and contributing to the onset and progression of the disease. T1DM, Type 1 diabetes mellitus. T2DM, Type 2 diabetes mellitus. GDM, Gestational diabetes mellitus. SCFAs, Short-chain fatty acids. BCAAs, Branched-chain amino acids. FFA, Free fatty acid. 2-HB, 2-Hydroxybutyrate. DAG, Diacylglycerol. AGEs, Glycation end-products. GABA, Gamma aminobutyric acid. GLP-1, Glucagon-like peptide-1. GIP, Glucose-dependent insulin-dependent polypeptide.

Gut microbiota is closely related to DM; glycated hemoglobin (HbA1c) levels are correlated with *Prevotella*, *Clostridia*, and *Ruminococcaceae*, and show a positive correlation with *Dorea*, *Bacteroidetes*, *Lactobacillus*, and *Bifidobacteria*. Further research has shown that *Rikenellaceae* and *Enterobacteriaceae* are positively correlated with hyperglycemia development, while *Synechococcus* sp., *Bifidobacterium adolescentis*, and *Chlorobium phaeovibrioides* are negatively correlated with IR [10]. Commensal bacteria support gut metabolism, while an increase in pathogenic bacteria can lead to dysfunction of the gut barrier, thereby affecting digestion and absorption [11]. *Clostridium* increases intestinal permeability by disrupting tight junction proteins in the gut, allowing lipopolysaccharides (LPSs) to enter the bloodstream and exert pro-inflammatory effects under conditions of high intestinal permeability [12]. *Escherichia coli*, *Klebsiella*, and *Desulfovibrio* can promote the production of LPSs, attacking pancreatic beta cells [13]. *Streptococcus* releases peptidoglycan and lipoteichoic acid, activating the NLRP3 inflammasome and enhancing the inflammatory response [14]. *Prevotella copri* (*P. copri*) is enriched in pregnant women with GDM and can elevate branched-chain amino acid (BCAA) levels, increasing the risk of developing GDM [15]. Despite numerous studies indicating that the gut microbiota is involved in the occurrence and development of DM, the specific mechanisms by which the microbiota affects different types of DM and its complications remain unclear. Existing research has confirmed that the gut microbiota plays a dual role in the production and regulation of metabolites such as short-chain fatty acids (SCFAs), BCAAs, lipopolysaccharides (LPSs), and indolepropionic acid (IPA). Dysregulation of metabolite expression can further exacerbate metabolic disorders. For example, low levels of SCFAs and high levels of BCAAs and LPSs may impair insulin signaling, leading to β-cell dysfunction, reduced glucose responsiveness, and insufficient insulin secretion, thus resulting in hyperglycemia (Figure 1). However, current research mainly focuses on a single type of DM, lacking comprehensive cross-type analysis.

Therefore, this review aims to extract experimental data on differences in gut microbiota abundance from the published literature, and to elucidate the unique characteristics of the gut microbiota in patients with different types of DM and its complications through a narrative review, and it attempts to construct the interaction relationships among gut microbiota, metabolites, and disease. Further evaluation of the potential effects of dietary adjustments, traditional Chinese medicine (TCM) treatments, and probiotics is conducted to provide new insights and therapeutic strategies for DM and its complications.

## 2. Characteristics of Gut Microbiota in DM and Its Complications

### 2.1. Gut Microbiota in T1DM

Gut microbiota plays a regulatory role through different abundances, and there is a significant correlation between the abundance of different intestinal microbiota and the incidence rate of T1DM. The *Veillonellaceae* is associated with a decreased susceptibility to T1DM, while the *Eubacterium coprostanoligenes group* significantly impacts T1DM complications. The external validation phase confirms that *Veillonellaceae* can reduce susceptibility to T1DM [16]. Mendelian randomization (MR) studies using the inverse-variance weighted method found that the *Saccharomyces* and *Bacteroides* have a causal relationship with T1DM [17]. The composition and abundance of gut microbiota vary with age in T1DM patients, exhibiting different disease characteristics. In adults with T1DM, the gut microorganisms *Blautia*, *Roseburia*, and *Faecalibacterium* that produce SCFAs are significantly reduced [18], and there is lower abundance of *Veillonella* [19]. Conversely, there is an increase in *Anaerobic Clostridia*, *Desulfovibrio*, *Ruminococcus*, *Bacteroides*, and *Lactobacillus johnsonii* [20]. Among middle-aged and elderly individuals, the relative abundance of *Bacteroidetes*, *Akkermansia*, and *Faecalibacterium* remains stable [21,22]. Children with T1DM exhibit unique microbial profiles, with increased levels of *Cyanobacteria*, *Fusarium*, *Bacteroides*, and the *Eubacterium hallii group* in the gut [23]. Although omics sequencing and statistical analysis indicate a close relationship between the gut microbiota and T1DM, there are no reports on the implementation of microbiota transplantation or supplementation in clinical diabetic patients. Streptozotocin is the primary research method for constructing diabetic animal models [24]. In animal model research, the imbalance between Th17 and Treg cells in the pancreas decreases after supplementation with *Akkermansia muciniphila* (*A. muciniphila*), and the upregulated Treg cells exert anti-inflammatory effects, thereby reducing islet damage [25,26]. The gut microbiota, in addition to regulating intestinal barrier functions and inflammation, also participates in the development of diabetes and its complications by modulating the host’s metabolite expression levels. Stroke is a common complication in patients with T1DM. *Faecalibacterium*, *Roseburia*, and *Blautia* are the main contributing bacteria for butyrate. Administration of butyrate to T1DM mice can regulate the response and polarization of BV2 cells and alleviate neural damage after middle cerebral artery occlusion by downregulating MyD88 [27]. Therefore, the gut microbiota plays a dual role in T1DM through immune cross-activation, barrier disruption, and metabolic regulation.

### 2.2. Gut Microbiota in T2DM

In T2DM, chronic hyperglycemia exacerbates the body’s inflammatory response, damages organ function, and leads to serious complications [28]. Compared to healthy individuals, T2DM patients exhibit elevated levels of *Clostridium*, *Sutterella*, *Dorea*, *Bacteroides*, *Eggerthella*, *Hungatella*, *Peptostreptococcus*, *Ruminococcus*, *Lactobacillus*, and *Parvimonas* [29,30]. Conversely, bacteria such as *Dehalobacterium*, *Anaeroplasma*, *Akkermansia*, *Roseburia*, *Adlercreutzia*, and *Oscillospira*, along with *Butyricicoccus*, *Lactobacillus*, *Bifidobacterium*, *Faecalibacterium*, and *Paraprevotella* are found in reduced abundance [29,31,32]. Certain bacteria, including *Roseburia*, *Bilophila*, *Oscillibacter*, and *Lactobacillus*, may serve as markers for T2DM [33], while *Flavonifractor* and *Haemophilus* are protective, and *Actinomyces* and *Candida* are risk factors [34]. Prediabetic individuals exhibit significant changes in anaerobic bacteria, including *Enterobacteriaceae*, *Prevotella 9*, *Blautia*, *Granulicatella*, and *Veillonella* [35]. These changes in the gut microbiota are closely associated with impaired glucose tolerance in prediabetes; for example, FGF15/19 inhibits hepatic gluconeogenesis and promotes glycogen synthesis, while a high abundance of *Bacteroides* downregulates the expression of the intestinal FXR signaling pathway, thereby reducing FGF15/19 levels, which may lead to hepatic IR and impaired glucose tolerance [36]. Moreover, animal models of T2DM play an important role in the study of disease treatment; the diabetic group shows significant increases in opportunistic pathogens such as *Eubacterium*, *Bilophila*, and *Mucispirillum*. Meanwhile, commensal bacteria like *Lactobacillus* were markedly reduced [37]. In T2DM mice, dapagliflozin promotes the production of GLP-1 by modulating the gut microbiota and tryptophan metabolism, thereby facilitating beta-cell regeneration and slowing the progression of DM [38,39]. Therefore, the gut microbiota may improve T2DM by regulating metabolites, intestinal barrier function, and insulin sensitivity [40].

### 2.3. Gut Microbiota in GDM

GDM is a type of DM first recognized during pregnancy [41]. GDM poses several risks to mothers, including gestational hypertension, preeclampsia, and preterm delivery. It may also lead to complications such as difficult labor, postpartum hemorrhage, and postpartum T2DM [42,43]. In women with GDM, the increase in estrogen, progesterone, and human placental lactogen during pregnancy leads to IR and pancreatic β-cell dysfunction [44]. After the birth of the fetus, the decrease in these hormone levels may temporarily restore compensatory balance of blood glucose, but the damage to pancreatic β-cells is usually irreversible. Under the influence of chronic inflammation and oxidative stress, IR and β-cell function impairment may further worsen, eventually leading to the development of T2DM [45]. It potentially impacts both maternal and fetal health. Compared to healthy individuals without GDM, GDM patients showed a decrease in *Lachnospiraceae*, *Blautia*, *Collinsella*, *Parabacteroides*, *Eubacterium hallii*, *Lactobacillus*, *Romboutsia*, and *Ruminococcus*, while *Bacteroides*, *Akkermensia*, *Acidaminococcus*, and *Lachnospiraceae NK4A136 group* showed a significant increase [46,47,48,49]. MR analysis links *Olsenella*, *Lachnoclostridium*, *Prevotella 9*, *Ruminococcus 2*, and *Oscillibacter* with GDM [50]. Oral probiotics can reverse these changes by altering the biosynthesis and metabolism of L-asparagine and L-aspartate, thereby alleviating the symptoms of GDM [51]. In patients with GDM, the levels of 2-Hydroxybutyrate (2-HB) and L-α-aminobutyrate are increased, whereas methionine sulfoxide and allantoin are significantly decreased. 2-HB not only indicates the loss of pancreatic function but also signifies IR [52]. *Ruminococcus*, *Eubacterium*, *Prevotella*, and *Parabacteroides* increase the synthesis of 2-HB [53]. Overall, the gut microbiota regulates the metabolic imbalance associated with GDM through metabolite interactions. Targeting specific microbiota for modulation holds promise as a potential therapeutic approach to alleviate GDM and offers significant research potential [54].

### 2.4. Gut Microbiota in DR

In healthcare institutions in Spain, the incidence of DR among patients with T2DM is 6.99 per 1000 person-years [55]. In patients with DR, levels of *Bacteroides*, *Roseburia*, *Lactobacillus*, *Ruminococcus*, and *Bifidobacterium* are elevated, whereas *Blautia*, *Faecalibacterium*, *Akkermansia*, *Clostridium*, *Romboutsia*, and *Escherichia-Shigella* are depleted [56,57,58]. MR studies show that *Peptococcaceae* and *Christensenellaceae* act as protective factors against DR, while *Ruminococcaceae*, *Adlercreutzia*, and *Eubacterium* increase the risk of DR [59]. Gut microbiome sequencing studies using a streptozotocin-induced diabetic rat model reveal similar microbiota profiles between diabetic rats and those with DR [60]. At the genus level, *Coriobacteriaceae*, *Veillonellaceae*, *Streptococcaceae*, and *Senegalimassilia* are significantly reduced, while *Burkholderiaceae*, *Fusobacterium*, *Pseudomonas*, and *Adlercreutzia* are significantly enriched [61,62]. Vascular endothelial growth factor (VEGF) promotes angiogenesis, increases vascular permeability, and induces inflammation and oxidative stress, thereby exacerbating the inflammatory response and microvascular damage in DR [63]. In the T2DM mouse model with hindlimb ischemia, VEGF signaling molecules were positively correlated with *Bifidobacterium*, *Clostridium sensu stricto 1*, *Lachnospiraceae NK4A136 group*, and *Coriobacteriaceae UCG 002*. On the other hand, *Lactococcus*, *Lachnoclostridium*, *Eubacterium brachy group*, *Kurthia*, *Weissella*, *Escherichia-Shigella*, and *Staphylococcus* were negatively correlated with VEGF signaling molecules [64].

### 2.5. Gut Microbiota in DKD

DKD is a severe complication of DM that primarily affects the kidneys, leading to a progressive decline in their function. DKD patients have higher abundances of *Blautia*, *Lactobacillus*, *Romboutsia*, *Turicibacter*, *Bacteroides*, *Akkermansia*, *Ruminococcus*, *Escherichia*, *Bilophila*, and *Bilophila* [65,66]. Conversely, the abundances of *Roseburia intestinalis*, *Lachnospiraceae*, *Faecalibacterium*, and *Prevotella* are significantly reduced [66,67]. Certain bacteria, such as the *Eubacterium nodatum group*, *Lactobacillus*, and *Faecalibaculum*, show a strong correlation with metabolic disorders in DKD [68]. *Rikenella* and *Bacilli* help decrease urinary protein levels, while *Lachnospiraceae* increases serum creatinine and indoxyl sulfate levels [69]. In DKD animal models, Lycoperoside H inhibits the abundances of *Turicibacter*, *Clostridium*, and *Bifidobacterium* while upregulating *Blautia*, exerting therapeutic effects and reducing DKD symptoms [70]. Metabolomics studies have revealed that DKD patients exhibit significantly higher levels of D-mannose, galacturonic acid, and citric acid. In contrast, levels of 3-methylindole, 3-(2-hydroxyethyl)indole, and indole propionic acid decrease, along with reductions in selenium metabolism and BCAA synthesis pathways [71].

### 2.6. Gut Microbiota in DN and DCD

DN and diabetic peripheral neuropathy (DPN) are both primarily characterized by nerve damage [72], leading to sensory and motor dysfunction. *Lachnospiraceae MK4B4 group*, *Parabacteroides*, and *Anaerotruncus* were reduced in DN patients [73]. Conversely, there is a notable increase in the abundances of *Enterococcus*, *Bifidobacterium*, *Allobaculum*, *Ruminococcaceae UCG 010*, *Anaeroplasma*, *Parasutterella*, *Dubosiella*, *Lactobacillus*, and *Turicibacter* [73,74]. MR studies have indicated that *Ruminococcaceae UCG 013* and *Eggerthella* are associated with a higher risk of developing DN [75]. In DM complicated by large vessel inflammatory lesions and hypertension, dietary phosphatidylcholine and L-carnitine are broken down by intestinal flora to produce trimethylamine N-oxide (TMAO) [76]. TMAO promotes vasoconstriction induced by angiotensin II, leading to hypertension [77]. Although oral antibiotics are a common treatment for inflammation, they reduce the beneficial effects of bacterial groups such as *Bacteroidetes* and *Clostridium* [78,79]. This reduction in gut diversity and alteration in microbial metabolic function can lead to decreased tryptophan levels and disrupted lipid metabolism, negatively impacting serum metabolism and worsening vascular sclerosis [80]. *Bacteroides*, *Lactobacillaceae*, and *Desulfovibrionaceae* are involved in lipid and glucose metabolism, and downregulating *Bacteroides fragilis* can slow atherosclerosis progression [81]. Twenty male patients with stable coronary artery disease consumed a daily beverage containing Lactobacillus reuteri 299v (Lp299v). The results indicated that Lp299v significantly reduced IL-8, IL-12, and leptin levels, and improved endothelial function [82].

### 2.7. The Gut Microbiome Is a Potential Target for DM Treatment

Expression levels of gut microbiota vary between different types of DM and its complications. For example, the abundance of *Faecalibacterium* is reduced in all DM and its complications (Table 1). *Ruminococcus* increases in conditions like T1DM, T2DM, DR, DKD, and DN. Regenerating islet-derived protein 3γ, a gut antimicrobial peptide with bactericidal activity, is effectively induced by *Ruminococcus gnavus* (*R. gnavus*), leading to rapid microbial death [83]. Implantation of a single strain of *R. gnavus* from lupus patients into C57BL/6 mice increased intestinal permeability [84]. Oral administration of *R. gnavus* in DN mice exacerbated renal lesions by upregulating IL-6, increasing levels of creatinine, urinary protein, and blood urea nitrogen levels. At the same time, it downregulated the tight junction proteins ZO-1, Occludin, and Claudin-1 [85]. In mice with gastric mucosal inflammation, *R. gnavus* led to plasma cell and lymphocyte infiltration into the gastric mucosa lamina propria, increasing the proportion of neutrophil infiltration [86]. Similarly, *Bacteroides* increases in both GDM, DR, DKD, and T2DM, while *Lactobacillus* rises in T1DM, T2DM, DR, DKD, and DPN, but decreases in GDM. *Bifidobacteria* decreases in T1DM, T2DM, and GDM but increases in DR and DN. Probiotic preparations containing *Bifidobacterium* and inulin (INU) have significantly and persistently reduced visceral fat area and total fat area in animals [87]. *Lactobacillus* exerts anti-inflammatory effects in the early stages of intestinal inflammation, protecting the intestinal barrier [88]. *Clostridium* decreases in both T1DM, GDM, and DR, and *Blautia* decreases in T1DM and DR but increases in GDM, DKD, and DN. *Roseburia* decreases in T2DM, GDM, and DKD, and *Romboutsia* decreases in T1DM, T2DM, GDM, DR, and DKD, while *Akkermansia* increases in DKD but decreases in T2DM, GDM, DR, and DN. We observed that the abundances of *Akkermansia*, *Clostridium*, and *Lactobacillus* at the genus level exhibited different trends. However, the microbial effects of *Akkermansia* at the species level were inconsistent [89], which might be due to the limitations of the current 16S rRNA sequencing technology. Moreover, factors such as genetics, diet, and chronic inflammatory stimuli may also contribute to these variations [90].

The gut microbiota plays a crucial role in the progression of DM and its complications, as well as in immune regulation [91]. In the T1DM model, following human amniotic mesenchymal stem cell treatment, the abundance of *Alcaligenes*, *Bifidobacterium*, and *Prevotella* increased, promoting the frequency of Th2 and Treg cells in the mesenteric lymph nodes, reducing the frequency of Th1 and Th17 cells, and significantly improving blood glucose and insulin secretion [92]. A study on human microbiota-associated mouse models found that transplanting fecal microbiota from T1DM patients and healthy individuals into germ-free mice resulted in phenotype reproducibility, providing feasibility and new research directions for microbiota transplantation [93]. A clinical controlled trial report shows that after receiving oral administration of *A. muciniphila*, T2DM patients experienced reduced weight and glycated HbA1c compared to the control group. Although the intergroup differences were not significant, this provides guidance for the clinical supplementation of gut microbiota [94]. As a new non-pharmacological treatment, the gut microbiota treats and improves DM and its complications by regulating gut permeability and insulin sensitivity, with significant potential clinical value [95]. By intervening in the abundance of these flora, the disease severity can be improved. These specific differences in gut microbiota are not only potential biomarkers for early detection and risk stratification but also provide promising therapeutic targets for modulating the gut microbiome to prevent or treat DM and its complications.

**Table 1:** Abundance and potential function of gut microbiota in DM and its complications.

Gut Microbiota	DM and Its Complications						Function	References
	T1DM	T2DM	GDM	DR	DKD	DN/DPN		
*Bacteroides*	↓	↓	↑	↑	↑	↓	Facilitates carbohydrate breakdown, improving insulin sensitivity and blood glucose regulation; dual inflammatory control	[29,47,56,65,96]
*Faecalibacterium*	↓	↓	↓	↓	↓	↓	Butyrate production, enhances gut barrier integrity and insulin sensitivity	[29,32,47,57,66,96]
*Lactobacillus*	↑	↑	↓	↑	↑	↑	Probiotics; maintaining intestinal homeostasis, regulating metabolism, and immunity; SCFA production	[29,48,57,66,74]
*Ruminococcus*	↑	↑	↓	↑	↑	↑	Generate acetic acid and butyric acid, dual insulin resistance, blood glucose and inflammation regulation	[20,28,47,58,66,96]
*Clostridium*	↓	↑	↓	↓	↑	↑	Butyrate production, improves insulin sensitivity, and lowers blood glucose; dual inflammatory control	[18,29,47,57,67,74]
*Blautia*	↓		↑	↓	↑	↑	Generate SCFAs, reduce inflammation, and improve insulin sensitivity and glucose metabolism	[18,46,56,67,96]
*Roseburia*	↑	↓	↓	↑	↓		Butyrate production, anti-inflammatory effects, and improves insulin sensitivity	[20,28,47,58,66]
*Romboutsia*	↓	↓	↓	↓	↑		Modulating gut barrier, glycemia, and inflammation	[29,49,56,67]
*Akkermansia*		↓	↓	↓	↑		Enhances gut barrier integrity and insulin sensitivity	[28,46,58,67]
*Bifidobacterium*	↓	↓	↓	↑		↑	Acetate and lactate production, improve glycemic control and insulin sensitivity	[29,46,57,73]
*Prevotella*	↓		↓		↓		SCFA production, dual insulin sensitivity, and glycemic control	[29,47,67]

T1DM, Type 1 diabetes mellitus. T2DM, Type 2 diabetes mellitus. GDM, Gestational diabetes mellitus. DR, Diabetic retinopathy. DKD, Diabetic kidney disease. DN, Diabetic neuropathy. DPN, Diabetic peripheral neuropathy. ↑, This abundance increases. ↓, This abundance decreased. SCFAs, Short-chain fatty acids.

## 3. Targeting the Gut Microbiota–Metabolite–Signaling Pathway Axis to Improve DM

### 3.1. Gut Microbiota Participates in DM by Regulating Metabolites

**Figure 2:**
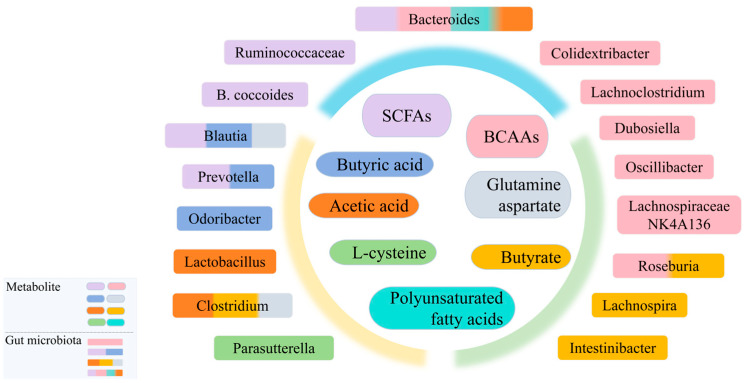
The gut microbiota plays a key role in the synthesis and metabolism of metabolites. As shown in the figure, the microbial community regulates the levels of specific metabolites, targeting one or more metabolites to influence the host’s metabolic balance. SCFAs, Short-chain fatty acids. BCAAs, Branched-chain amino acids.

The gut microbiota not only plays a key role in digestion and nutrient absorption but also engages in complex interactions with the host’s metabolic pathways through its metabolites, thus affecting the expression levels of multiple systems within the body, including energy metabolism, fat metabolism, glucose metabolism, and immune response [97]. However, the specific mechanisms remain unclear. Studies have shown that certain bacteria, such as *Lactobacillus* and *Prevotella UCG 001*, are closely associated with sphingolipid metabolism as well as the biosynthesis of tryptophan and tyrosine [98]. *Blautia* and *Clostridium* are positively linked to glutamine levels, while showing negative correlations with aspartic acid, methionine, lysine, and 1-methyl-L-histidine [99]. On the other hand, *Alloprevotella* is inversely related to γ-aminobutyric acid and hexanoylglycine, whereas *Prevotella* is positively correlated with compounds like 5-hydroxyindoleacetic acid, O-acetyl-L-serine, glutamic acid, and aspartic acid [100]. Furthermore, the families *Christensenellaceae R7* and *Ruminococcaceae UCG 002* are strongly linked with higher expression of ornithine and L-arginine [101]. L-isoleucine and phenylalanine are positively correlated with *Enterobacteriaceae*, *Clostridiaceae*, and *Romboutsia* [102]. These complex interactions between microbiota metabolites underscore the critical role the gut microbiota plays in regulating metabolic processes. Specific microbiota may exert bidirectional effects in the pathogenesis of metabolic diseases by regulating fatty acid, amino acid, and lipid metabolism pathways (Figure 2).

Previous studies have confirmed that balancing SCFAs is beneficial for the human body. *Oscillibacter*, a producer of LPSs, along with *Bacteroides* and *Odoribacter*, which produce SCFAs, are less abundant in DKD patients [71]. *Blautia coccoides* (*B. coccoides*), *Prevotella*, along with *Ruminococcaceae* and *Lachnospiraceae*, are involved in producing SCFAs [103]. *Clostridium sensu stricto*, *Lactobacillus*, and *Bacteroides* primarily produce acetic acid [104]. Additionally, combined interventions of *Veillonella* and *Lactobacillus* increased the abundance of commensal bacteria such as *Akkermansia* and the total content of SCFAs, while reducing the abundance of opportunistic pathogens like *Desulfovibrio* and *Escherichia-Shigella*. These changes collectively exerting anti-inflammatory effects [105]. In mice with T2DM, anaerobic bacteria such as *Bacteroides*, *Cyanobacteria*, *Colidextribacter*, *Lachnospiraceae NK4A136*, *Roseburia*, *Dubosiella*, *Oscillibacter*, and *Lachnoclostridium* are involved in the biosynthesis of BCAAs [106]. In diet-induced obese mice, gavage administration of *Bacteroides spp*. improved imbalanced BCAA metabolism and reversed the increased body weight [107].

In an inflammatory bowel disease mouse model, *Fournierella*, *Clostridium*, and *Peptostreptococcus* promote the synthesis of IPA, while *Lachnoclostridium*, *Erysipelatoclostridium*, and *Parabacteroides* reduce its abundance by consuming IPA [108]. Clostridium species promote the synthesis of IPA, exerting inhibitory effects that significantly improve glucose metabolism and reduce body weight [109]. TMAO has regulatory effects on insulin sensitivity, inflammatory response, and lipid metabolism [110]. *Bacteroides* and *Clostridium* promote the production of trimethylamine N-oxide [111]. Quinic acid modulates the abundance of gut microbiota by downregulating Streptococcus danieliae and upregulating Ileibacterium valens and Lactobacillus intestinalis. It regulates TMA/TMAO-mediated hepatic lipid metabolism, improving atherosclerosis in Apoe^−/−^ mice [112]. 16S sequencing results of the gut microbiota in patients with impaired glucose tolerance show a decrease in *Faecalibacterium*, which is capable of producing taurodeoxycholic acid and carnosine. This reduction weakens the inhibitory effect on insulin-like growth factor binding protein 3, exacerbating hyperglycemia and related vascular lesions [113]. Therefore, the gut microbiota demonstrates new research potential by reshaping SCFAs, BCAAs, and other regulatory metabolites to regulate metabolic diseases.

### 3.2. BCAAs, SCFAs, and IPA Regulate Signaling Pathways to Improve DM and Its Complications

**Figure 3:**
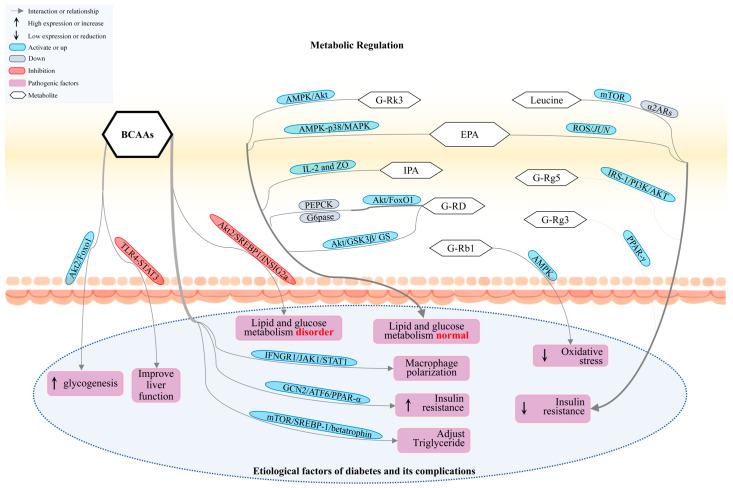
Metabolites’ involvement in DM and its complications through the regulation of signaling pathways. Metabolites such as BCAAs, ginsenosides, and IPA modulate signaling pathways including AMPK, Akt, and ROS, thereby influencing the onset and progression of various diseases. BCAAs, Branched-chain amino acids. G-Rk3, Ginsenoside Rk3. EPA, Eicosapentaenoic Acid. IPA, Indolepropionic acid. G-Rg5, Ginsenoside Rg5. G-RD, Ginsenoside Rd. G-Rg3, Ginsenoside Rg3. G-Rb1, Ginsenoside Rb1.

The insulin signaling and *AMPK* pathways are primarily responsible for glucose uptake, glycogen storage, and endocrine metabolism [114]. Excessive activation of the mTOR pathway, however, can exacerbate IR [115]. The NF-κB pathway is closely related to the body’s inflammation levels. High expression of NF-κB leads to a high inflammatory response in DM, promoting the occurrence of complications [116]. Additionally, the binding of advanced glycation end-products (AGEs) to their receptor for advanced glycation end-products (RAGE) initiates a series of downstream signals that contribute to the development and progression of DM. Studies have confirmed that different expression levels of metabolites can regulate DM-related signaling pathways. BCAAs, significant components of AAs in the body, regulate the Akt2 signaling pathway, impacting lipid metabolism and glucose homeostasis [117] (Figure 3). Supplementing BCAAs may activate the INFGR1/JAK1/STAT1 pathway, promoting pro-inflammatory macrophages and IR [118]. BCAAs enhance the fatty acid oxidation function by modulating the GCN2/ATF6/PPAR-α signaling pathway [119]. The liver plays a crucial role in regulating diabetes homeostasis. In a cirrhotic rat model treated with BCAAs, BCAAs reduced the protein expression of lipopolysaccharide-binding protein, Toll-like receptor 4, and STAT3, thereby protecting liver function [120]. However, in T1D mice, the gut microbiota shows insufficient degradation of BCAAs. This activates the mTOR signaling pathway, leading to mitochondrial damage and cardiomyocyte apoptosis [121].

**Figure 4:**
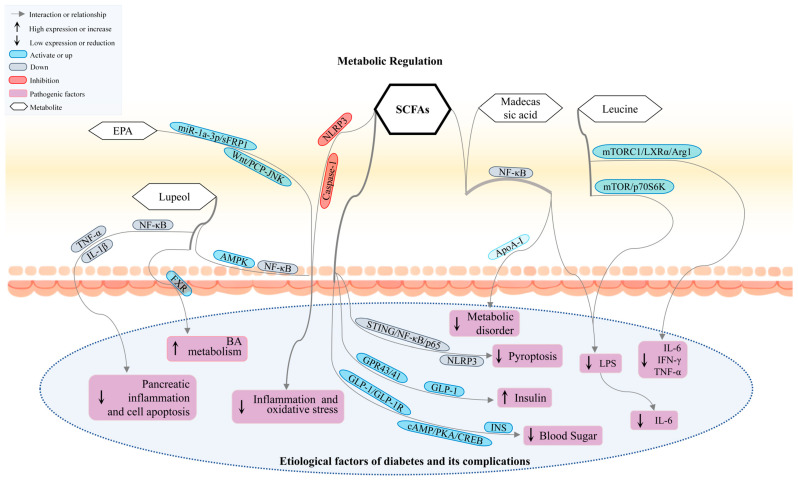
Metabolites like SCFAs, EPA, leucine, and lupeol play significant roles in the development of DM and its complications by regulating key signaling pathways, including NF-κB, AMPK, and mTOR. These pathways are essential for maintaining metabolic homeostasis. EPA, Eicosapentaenoic Acid. SCFAs, short-chain fatty acids.

Research has found that *Streptococcus*, *Prevotella*, and *Faecalibacterium*, along with species like *Bacteroides* and *Eubacterium*, are significantly reduced [122,123], leading to decreased SCFA production in GDM patients. In diabetic neuropathy, SCFAs exert antioxidant protection through the G protein-coupled receptors (GPR)43 pathway (Figure 4); under oxidative stress induction, neuronal cell lines undergo dysfunction, whereas GPR43 exerts a protective effect by inhibiting H_2_O_2_-induced neuronal damage [124]. Additionally, the NF-kappaB/MAPKs signaling pathway further reduces inflammation in the body under the inhibition of GPR43 [125]. The NLRP3/Caspase-1 pathway regulates THP-1 cell metabolism inhibited by SCFAs to alleviate cellular inflammation [126]. The GLP-1/GLP-1R/cAMP/PKA/CREB/INS pathway enhances the management of T2DM under the influence of SCFAs [127]; SCFAs inhibit the HDAC3-H3K27ac-PPAR-γ axis, thereby reducing lipid storage in the body [128]. Additionally, SCFAs decrease NF-κB transcriptional activity and restore apolipoprotein A-I transcription levels in HepG2 cells [129]. Butyrate reduces apoptosis, thereby ameliorating DKD, primarily by inhibiting the NLRP3 inflammasome via the STING/NF-κB/p65 pathway [130]. Restoration of gut microbiota and immune regulation imbalance was observed in T1DM patients receiving oral SCFAs biotherapy. Transplanting the fecal microbiota from these patients into humanized GF mice could delay the progression of DM [131]. Therefore, restoring the balance of SCFA expression levels can further alleviate DM and its complications. T2DM is often accompanied by cognitive decline. Studies have shown that IPA can prevent neuronal death and restore mitochondrial function. Under intermittent fasting treatment, the levels of SCFAs and IPA are upregulated in db/db mice. However, antibiotic treatment exacerbates cognitive impairment by inhibiting gut microbiota and reducing IPA [132]. Supplementation with IPA restored colonic barrier function by upregulating IL-25 and alleviated obesity and metabolic disorders induced by a high-fat (HF) diet [133]. Furthermore, network pharmacology studies have revealed that IPA can modulate signaling pathways such as NF-κB, VEGF, and TNF. These pathways are closely associated with DM and its complications [134,135,136].

### 3.3. Other Metabolite Regulation Pathways Improve DM and Its Complications

The PI3K/AKT/mTOR, NF-κB, and MAPK signaling pathways, along with oxidative stress, play crucial roles in diabetes and its complications by regulating glucose metabolism and insulin sensitivity. In DM rats, TMAO accelerates wound healing by modulating the PI3K/AKT/mTOR pathway [137]. Increased expression of neutrophil extracellular traps (NETs) can inhibit the function and angiogenesis of HTR-8/Svneo cells in patients with GDM. However, feeding PAD4^−/−^ mice with TMAO inhibits NET formation, thereby promoting the development of the placenta and fetus [138]. In other metabolites such as phytosphingosine, the highly inflammatory response induced by pathogenic cytokines is improved through the MAPK and NF-κB pathways [139]. High inflammatory response mediated by LPSs can be reversed by the inhibition of NF-κB and downstream pathways by madecassic acid [140]. On the other hand, lupeol alleviates oxidative stress-induced islet inflammation and apoptosis in streptozotocin-induced hyperglycemic mouse models [141]. Ginsenosides exhibit unique biological effects in DM metabolism, such as ginsenoside Rk3 improving metabolic disorders by upregulating the AMPK/Akt pathway [142]. Ginsenoside Rg3 exerts cardioprotective effects by activating the PPAR-γ signaling pathway [143]. Ginsenoside Rd alleviates hyperglycemia complications through the Akt pathway [144], and ginsenoside Rb1 mitigates diabetic atherosclerosis via the AMPK pathway [145]. IR in DM can be improved by ginsenoside Rg5 upregulating the Sirt1/PGC-1α pathway [146].

HepG2 cells have a regulatory effect on IR, and eicosapentaenoic acid (EPA) can reverse the imbalance of HepG2 cells through the ROS/JUN pathway [147] and alleviate inflammation and oxidative stress by miR-1a-3p/sFRP1/Wnt/PCP-JNK [148]. Furthermore, EPA activates the AMPK pathway to optimize glucose metabolism [149], and promotes the generation of docosahexaenoic acid, providing antioxidant protection in retinopathy [150]. Chenodeoxycholic acid has multiple biological effects in the human body by upregulating ROS/p38 MAPK/DGAT1 to catalyze lipid peroxidation in DM [151]. Chenodeoxycholic acid can also activate the FXR-MLCK pathway, further reducing LPS damage to the intestinal epithelial barrier [152]. In addition, the AMPK/NF-κB and TGFβ1-Nrf2 pathway can promote ferritin deposition and cell apoptosis, while Lupeol exerts anti-inflammatory effects by inducing oxidative stress and apoptosis through the AMPK/NF-κB pathway [153], further improving bile acid metabolism [154]. We believe that targeting the gut microbiota, metabolites, and disease-related signaling pathways within the “gut microbiota–metabolite–signaling pathway” network can restore metabolic balance in diabetic patients, thereby potentially improving DM management and reducing complications (Table 2).

## 4. TCM Therapeutic Strategies Targeting Gut Microbiota and Metabolites

### 4.1. TCM Treats DM Through Gut Microbiota and Metabolites

Gut microbiota and their metabolites have become new avenues for TCM treatment [155]. Polysaccharides and polyphenolic compounds in TCM regulate gut microbiota and their metabolites, reduce inflammatory responses, and improve gut health. Additionally, the bioactive components of TCM can be further activated by the gut microbiota, enhancing pharmacological activity. TCM regulates SCFA expression levels through gut microbiota, controlling hyperglycemia and reducing inflammation (Table 3). In T1DM, supplementation with extra virgin olive oil can slow gastric emptying, enhance the abundance of *Lachnoclostridium* and *Ruminococcaceae UCG 005*, and promote the production of SCFAs [156]. Additionally, prebiotic ham combined with high-amylose maize starch improves beta-cell function and blood glucose regulation through SCFA modulation [157]. Astragalus polysaccharide can increase the levels of propionic acid, acetate, and butyrate salts in T1D-diabetic mice, thus helping to restore the gut microbiota and suppress the inflammatory response [158]. INU and soluble fiber inulin and omega 3-PUFA promote the production of essential SCFAs by commensal bacteria, which helps regulate the immune system by recruiting regulatory T cells to the pancreas via CCL17, enhancing the immune response [159]. TCM has shown beneficial effects in the treatment of T2DM. Fuzhuan brick tea increases *Ruminococcus*, lactic acid bacteria, and *Lachnospiraceae NK4A136 group*, while reducing *Prevotella* and *Faecalibacterium*, thereby enhancing SCFA levels [160]. Phellinus linteus enhances the levels of *Bacteroides*, *ParaBacteroides*, and *Alistipes*, which are associated with SCFAs and bile acid metabolism [161]. Canagliflozin upregulates *Muribaculaceae* and *Bacteroidaceae*, promoting an increase in SCFA levels [162]. Carvacrol restored blood glucose and lipid disorders in T2DM rat models by increasing SCFA levels and upregulating GPR 43/41 expression [163]. In a GDM mouse model, high-fermentation dietary fiber like konjac reduces intestinal permeability and LPS by increasing abundances of *Lachnospiraceae* and butyrate [164].

The study of TCM extracts has a long history in China. Cinnamaldehyde is the main component of the cinnamon tree. In T1D mice, blood glucose levels are regulated by cinnamaldehyde through modulation of the gut microbiota and bile acids, leading to a reduction in blood glucose [165]. In T2DM mice, mulberry leaf water extract restores intestinal permeability and glucose and lipid metabolism, and this effect is achieved through the gut microbiota [166]. Jiang-Tang-San-Huang promotes the abundance of *Bacteroides* and *Bifidobacteria*, which helps to correct gut dysbiosis and regulate inflammatory pathways [167]. Polysaccharides from Dendrobium officinale boost the abundance of *Eubacterium*, *Bifidobacterium*, and lactic acid bacteria, inhibit *Helicobacter pylori*, and significantly enhance the intestinal barrier function [168]. Components of Tribulus terrestris help regulate imbalanced microbiota and increase the levels of commensal bacteria [169]. Ganzhou orange peel pectin, a plant extract, reduced opportunistic pathogens like *Alistipes*, *Oscillibacter*, and *Helicobacter* in diabetic mice, while commensal bacteria like *Dubosiella*, *Akkermansiaceae*, and *Atopobiaceae* increased [170].

Polysaccharides have become a prominent focus in TCM [171]. Sequencing of the gut microbiota in a T1D rat model revealed that the polysaccharides of D. huoshanense promote the abundance of Firmicutes and the expression levels of SCFAs [172]. Crude polysaccharides from D. divaricata regulate the levels of mucin-2 and tight junction proteins, restoring intestinal function, and enhance the regulatory role of insulin receptor substrate-1 on insulin [173]. Licorice-derived polysaccharides administered to mice increased levels of *Romboutsia*, *Akkermansia*, lactic acid bacteria, and other commensal bacteria, while inhibiting the pathogenic bacterium *Bacteroides* [174]. Superoxide dismutase and catalase, under the action of golden flowers containing polysaccharides, polyphenols, proteins, and amino acids, downregulate the expression of TNF-α, IL-4, and IL-6 to alleviate oxidative stress in the body [175]. Modern technology has also facilitated the discovery of traditional medicine. For example, low-methoxyl pectin opens up a realistic option for controlling gut microbiota and preventing T1DM [176]. Flavonoids from cactus upregulate the expression of SCFAs to exert resistance against DKD [177].

**Table 3:** Regulatory effects of TCM on gut microbiota and metabolites in different types of DM.

Types	Drug	Research Subject	Gut Microbiota		Metabolites		References
			Increase	Decrease	Up	Down	
T1DM	Extra virgin olive oil	NOD mice	*Lachnoclostridium*, *Ruminococcaceae UCG 005*	*Lachnospira*, *Eubacterium*	Madecassic acid, Lupeol	Ginsenoside, Oleamide	[156]
	Astragalus polysaccharides	T1D mice	*Muribaculum*, *Lactobacillus*, *Bacteroides*	*Corynebacterium*, *Brevibacterium*, *Brachybacterium*	Acetic acid, PA, BA, SCFAs	Isobutyric acid, Isopentanoic acid, BCFAs	[158]
	Soluble fiber inulin and omega 3-PUFA	NOD mice	*Akkermansia*	*Bacteroides intestinalis*, *Streptococcus* sp.	Docosapentaenoic acid, Docesahexaenoic acid, Eicosapentaenoic acid	2-Hydroxybutyric acid	[159]
	Cinnamaldehyde	T1DM model mice	*Parasutterella*, *Odoribacter*, *Burkholderiales*	*Dorea*, *Mucispirillum*	Myristoleic acid, 3-hydroxybutyric acid	Hydrocinnamic acid, 2-phenylpropionate	[165]
	Polysaccharides of *D. huoshanense*	T1D mice	*Lactobacillus*, *Megasphaera*	*Bacteroides*, *Parabacteroides*, *Dorea*, *Enterocloser*	Acetic acid, PA, Butyrate	/	[172]
	Crude polysaccharides	T1D mice	*Lactobacillus*	*Ruminococcaceae*, *Lachnospiraceae*, *Rikenellaceae*	/	/	[173]
	“Golden-flower” Tibetan tea	T1D mice	*Lactobacillus*, *Lachnospiraceae NK4A136 group*	*Bacteroides*	SCFAs, Superoxide dismutase, Catalase	/	[175]
	Low-methoxyl pectin	NOD mice	*Firmicutes*, *TM7*, *Proteobacteria*	*Bacteroidetes*	Cetate, Propionate, Butyrate, SCFAs	/	[176]
T2DM	Ethanol extract of propolis	T2D mice	*Parasutterella*, *Bifidobacterium*, *Faecalibaculum*, *Dubosiella*, *Lachnoclostridium*		N-Acetyl-L-glutamic acid, D-(+)-Galactose, (R)-Lactate, L-(+) Lactic acid	Lactulose, L-Proline, O-Acetyl-L-serine, S-Adenosyl-L-homocysteine	[37]
	Polysaccharides from Phellinus linteus	T2D rat model	*Alistipes*, *Prevotellaceae*, *Bacteroides*, *Parabacteroides*	*Faecalibaculum*, *Lachnospiraceae*	SCFAs, Primary bile acids	Aspartate aminotransferase alanine aminotransferase, Primary bile acids	[161]
	*Morus alba* L. water extracts	T2D mice	*Dubosiella*	*Anaerovorax*, *Bilophila*, *Blautia*, *Lachnoclostridium*	Branched-chain ketoacid, Dehydrogenase E1α	Amino acid	[166]
	Jiang-Tang-San-Huang	T2D rat model	*Romboutsia*, *Lactobacillus*, *Bacteroides*, *Bifidobacterium*	*Enterococcs*	Primary bile acids, Chenodeoxycholic acid	Taurocholic acid	[167]
	Navel orange peel pectin	diabetic mouse	*Dubosiella*, *Akkermansia*, *Lachnospiraceae*, *Atopobiaceae*	*Muribaculaceae*, *Lachnospiraceae NK4A136 group*	Acetic acid, Total acid, BA	PA	[170]
	Polysaccharide extract	T2D mice	*Akkermansia*, *Lactobacillus*, *Alistipes*, *Romboutsia*, *Faecalibaculum*	*Bacteroides*, *Alloprevotella*, *Escherichia-Shigella*, *Clostridium*	Propionate, Butyrate	Triglycerides, Total cholesterol	[174]
GDM	Inulin-type fructans	GDM mice	*Akkermansia*, *Bifidobacterium*	*Dubosiella*	BA, Acetic acid	/	[122]
	Konjac	GDM mice	*Dubosiella*, *Monoglobu*	*Bavteroides*, *Romboutsia*, *Faecalibaculum*	Phenylalanine	Diamine oxidase, LPS, Valine, Leucine, Isoleucine	[164]

T1DM, Type 1 diabetes mellitus. NOD mice, Non-obese diabetic mice. T2DM, Type 2 diabetes mellitus. GDM, Gestational diabetes mellitus. PA, Propionic acid. BA, Butyric acid. BCFAs, Branched-Chain Fatty Acids. SCFAs, Short-chain fatty acids.

### 4.2. TCM Treats DM Complications Through Gut Microbiota and Metabolites

DM complications seriously threaten human health. The Luo Tong Formula improves intestinal microecological balance in diabetic rats by reversing changes in gut microbiota, such as *Enterobacteriaceae*, *Bacteroidetes*, *Prevotellaceae*, *Enterococcaceae*, and *Klebsiella*, thereby mitigating the hyperglycemic and inflammatory state of DR [178]. Quercetin improves DR by balancing gut microbiota and intestinal permeability [179]. In the DKD mouse model, the Qing-Re-Xiao-Zheng formula inhibits the expression of NF-κB and Toll-like receptor 4 [180]. The polysaccharides in the Fufang-zhenzhu-tiaozhi formula and polysaccharides from Moutan Cortex increased the expression levels of short-chain fatty acids, thereby alleviating renal injury [181,182]. Fructooligosaccharides, a type of soluble dietary fiber, act as prebiotics that alleviate DKD by preventing LPSs from entering the circulatory system and reducing their expression levels within the body [183]. *A. muciniphila* and *Lactobacillus murinus* are closely related to glucose and lipid metabolism. Astragalus membranaceus and Salvia miltiorrhiza improve the DKD rat model by upregulating the abundance of *A. muciniphila* and *Lactobacillus murinus* [184]. INU-type fructans enhance the abundance of *Akkermansia* and *Candidatus Saccharimonas*, thereby alleviating mitochondrial dysfunction and downregulating toxic glucose metabolites, which further reduce glomerular injury and renal fibrosis [185]. These microbial communities significantly regulate metabolite levels in the body, thereby treating and alleviating DKD (Table 4).

In DN, corn silk polysaccharides improve DN by adjusting the abundance of *Dubosiella*, *Bacteroidetes*, and *Firmicutes*, enhancing the synthesis and metabolism of bile acids, FAs, tryptophan, and tyrosine [186]. San-Huang-Yi-Shen Capsules affect the abundance of *Ruminococcaceae UCG 005*, *Lactobacillus*, *Anaerostipes*, and *Anaerococcus*, and affect the expression levels of metabolites such as L-carnitine and threonine [187]. *Clostridium*, *Bifidobacterium*, and *Fusobacterium* inhibit p-cresol formation, while magnesium lithospermate B and its metabolite danshensu improve DN via gut microbiota [188]. In animal models, sanziguben polysaccharides reduce the abundance of *Proteobacteria* and *Klebsiella*, thereby modulating LPS levels in DN mice. TCM also has therapeutic effects on DPN. In the DPN rat model, Jinmaitong restores gut microbiota imbalance and upregulates serum NRG1 levels to protect against DPN [189]. Similarly, gingerol-enriched ginger and quercetin also have preventive and therapeutic effects in DPN [190,191]. Agarotetraose increased the abundance of *Muribaculaceae*, *Lachnospiraceae*, and *Blautia*, while decreasing the abundance of *Faecalibaculum* and *Desulfovibrio*. Further studies showed that the total bile acid content in feces is negatively correlated with atherosclerosis progression [192]. Huangqi Guizhi Wuwu Decoction has a significant therapeutic effect on diabetic peripheral neuropathy by regulating the abundances of *Lactobacillus*, *Prevotella*, *Bacteroidetes*, and *Desulfovibrio*. Correlation analysis has suggested that it may regulate the biosynthesis of unsaturated FAs and the metabolic functions of valine, leucine, and isoleucine [193]. Berberine is a prominent topic in pharmacological research [194]. Animal studies have found that berberine regulates the *Lachnospiraceae NK4A136 group*, *Eubacterium*, and *Bacteroidales S24 7 group*, inhibiting the production of trimethylamine and trimethylamine N-oxide, thereby alleviating choline-induced atherosclerosis.

**Table 4:** Regulatory effects of TCM on gut microbiota and metabolites in different types of DM complications.

Types	Drug	Research Subject	Gut Microbiota		Metabolites		References
			Increase	Decrease	Up	Down	
DR	Luo Tong formula	DR rat model	*Candidatus_Saccharimonas*, *Romboutsia*, *Enterorhabdus*	*Prevotella*	/	/	[178]
	Quercetin	Sprague Dawley mice	*Turicibacter*, *Roseburia*, *Bifidobacterium*	*Streptococcus*, *Veillonella*, *Prevotella*	Acetic acid, PA, BA	/	[179]
DKD	Tangshen Formula	DKD mice	*Barnesiella*	*Romboutsia*, *Akkermansia*, *Collinsella*	Tryptophan, 5-hydroxyindoleacetate, Glutamic acid, Aspartate	Indole-3-acetic acid, Xanthurenic acid	[100]
	Qing-Re-Xiao-Zheng formula	DKD mice	*Rikenellaceae*, *Akkermansia*	*Desulfovibrio*	SCFAs	LPSs	[180]
	Fufang-zhenzhu-tiaozhi formula	DKD mice	*Bacteroidota*, *Actinobacteriota*, *Pseudonocardia*	*Weissella*, *Enterococcus*, *Akkermansia*	PA, Methylmalonic acid, Butanoic acid	3-hydroxybutyrylcamitine, Gamma-muricholic acid	[181]
	Polysaccharides	DKD rat model	*Mollicutes*, *Bacteroidota*, *Ruminococcaceae_UCG-014*	*Lactobacillus*	Acetic acid, PA, BA	Isovaleric acid, BCFAs	[182]
	Salvia miltiorrhiza	DKD rat model	*Akkermansia*, *Lactobacillus*, *A. musciniphila*	*Prevotellaceae UCG 001*	Phytosphingosine, Sphinganine	Indolyl sulfate, P-cresolsulfate, Myo-inositol	[184]
DN	San-Huang-Yi-Shen capsule	DN rat model	*Lactobacillus*, *Allobaculum*, *Ruminococcaceae UCG 005*, *Anaerovibrio*, *Bacteroides*	*Candidatus Saccharimonas*	Amino sugar, Pyruvate metabolism, Nucleotide sugar metabolism	TCA cycle, Arachidonic acid, Mannose metabolism	[187]
	Magnesium lithospermate B	diabetic mouse	*Bifidobacterium*, *Lachnospiraceae*, *Aerococcus*, *Bacteroidales*	*Alistipes*, *Lachnospiraceae NK4A136 group*	BA, Isobutyric acid, Pentanoic acid, Alanine, Threonine, Glycine, Lysine	Tyrosine	[188]
DPN	Jinmaitong	DPN rats	*Helicobacterae*, *Blautia*, *Escherichia-Shigella*	*Clostridium*, *Oscillibacter*	/	/	[189]
	Quercetin	DPN rats	*Prevotella*, *Escherichia-Shigella*, *Bifidobacterium*	*Desulfovibrio*, *Lactobacillus*	/	/	[190]
	Gingerol-enriched ginger	DPN rats	*Lachnospiraceae*		/	/	[191]
	Huangqi Guizhi Wuwu Decoction	db/db mice	*Lactobacillus*, *Alloprevotella*, *Bacteroides*	*Lachnoclostridium*, *Blautia*, *Desulfovibrio Ruminococcus*, *Akkermansia*, *Caproiciproducens*	/	Sphinganine, Sphingosine 1-phosphate, Phytosphingosine	[193]

DR, Diabetic retinopathy. DKD, Diabetic kidney disease. DN, Diabetic neuropathy. DPN, Diabetic peripheral neuropathy. PA, Propionic acid. BA, Butyric acid. BCFAs, Branched-Chain Fatty Acids. SCFAs, Short-chain fatty acids. LPSs, Lipopolysaccharides.

### 4.3. Future Treatment Strategies of TCM in DM

TCM exerts unique therapeutic advantages in improving glucose and lipid metabolism and alleviating inflammation through multi-target modulation of gut microbiota and its metabolites [184]. Due to their genetic similarity to humans, rodent models, particularly mice [195], are widely used in preclinical studies of TCM for the treatment of DM. Researchers have developed various modeling strategies to induce acute or progressive β-cell destruction, hyperglycemia, and IR in these animals. For instance, Kachapati et al. [196] and Sharma et al. [197] found that mice exhibit many characteristics similar to humans regarding hyperglycemia and renal pathology. However, there are significant physiological and metabolic differences between rodents and humans. These differences include variations in lifespan, immune system responses, and metabolic rates, which may affect the translation of research findings to human clinical applications. Nevertheless, rodent models remain important tools for advancing our understanding and treatment of DM. Liu et al. [198] and Takaichi et al. [199] successfully transplanted human stem cells into DM models induced in C57BL/6J mice without encountering issues of immune rejection in transplantation therapy. Therefore, it is important to critically analyze TCM research findings based on animal models.

TCM mainly includes herbal medicine, acupuncture, dietary therapy, and physical exercise. Among these, Chinese herbal medicine is the most commonly used [200]. However, Chinese herbal medicine involves potential risks, such as the use of toxic substances, ingredients from endangered species, and poorly characterized bioactive compounds [201]. Therefore, the use of herbal medicine outside the framework of TCM diagnosis is not considered a standardized medical procedure. Although TCM has shown significant protective effects against diseases such as DM, there is a lack of critical analysis and standardized reporting to guide its use [202]. Therefore, it is essential to rigorously evaluate the role of TCM in nutritional intervention and precision medicine when exploring the effectiveness of TCM and its components in improving and treating DM and its complications [203]. Although the mechanisms of bioactive components such as polysaccharides remain unclear, we believe that future treatment strategies for DM and its complications should focus on regulating specific microbiota, enhancing intestinal barrier function [204], and reducing the entry of harmful bacteria and LPSs into the bloodstream, thus mitigating metabolic inflammation [205]. Furthermore, TCM can modulate metabolites such as SCFAs, BCAAs, bile acids, and TMAO, improving insulin sensitivity, microvascular complications, and glucose homeostasis. It also exerts anti-inflammatory effects by inhibiting the activity of inflammatory factors like TNF-α, IL-6, and signaling pathways such as *NF-κB* and *AGEs/RAGE* [206]. Targeting multiple points in the “gut microbiota–host metabolite” network to conduct clinical translational research on TCM derivatives (such as TCM dietary therapy, medicinal herbal beverages, and herbal medicine extracts) in the treatment of DM and its complications may become a promising new treatment approach.

## 5. Diet as Medicine: Treatment Strategies for DM and Its Complications

### 5.1. Adjust Dietary Structure to Improve DM and Its Complications

Eating habits exhibit regional, cultural, and ethnic characteristics; dietary intake is broken down and digested with the help of gut microbiota. The Mediterranean diet (MedDiet) is characterized by an increased intake of legumes, fruits, whole grains, and vegetables, and reduced intake of red meat and sweets [207]. In MedDiet interventions involving increased physical activity and reduced caloric intake (Table 5), the *Eubacterium hallii group* and *Dorea* decreased, while alpha diversity significantly increased [208]. The Japanese diet is similar to the MedDiet, with a higher intake of vegetables, fish, and soy products, and a lower intake of meat. This diet leads to an increase in *Lachnospiracea incertae sedis*, *Gemmiger*, *and Faecalibacterium*, while SCFA-producing bacteria significantly decline [209]. Time-restricted eating encourages regular eating patterns, whereas Ramadan fasting involves a reversal of typical eating times. Significant differences in the abundances of *Roseburia*, *Akkermansia*, *Bacteroides*, *Prevotella* 9, and *Megamonas* were observed between these two dietary patterns [210]. Floral tea is commonly part of daily life. Feeding mice an HF and high-sucrose (HS) diet along with alcohol increased opportunistic pathogens; however, supplementation with water extract of *Chrysanthemum morifolium* Ramat. reversed this effect, significantly increasing the abundances of *Clostridium* and *Faecalibaculum* [211]. Capsaicin is commonly present in Chinese cuisine. Studies have found that capsaicin can reduce the abundance of *Streptococcus*, *Enterococcus*, *Barnesiella intestinihominis*, and *Eubacterium uniformis*, which are closely related to the generation of SCFAs [212].

**Figure 5:**
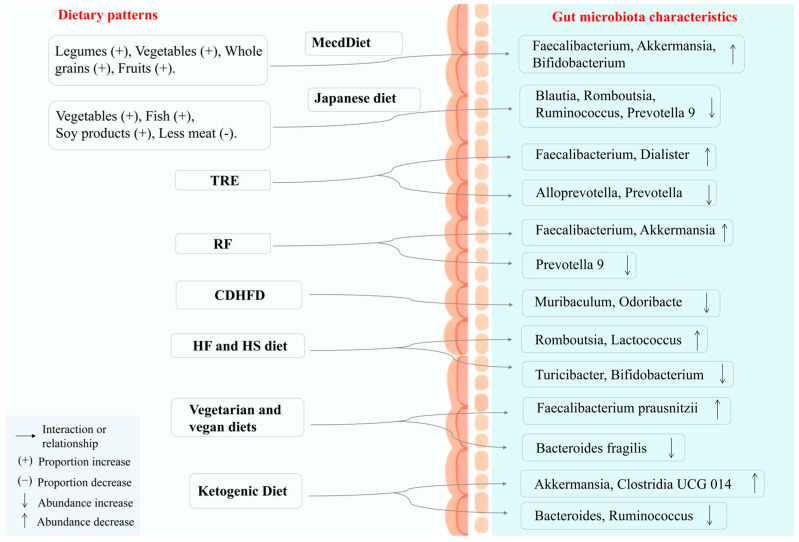
Different dietary structures and eating habits have a regulatory effect on the gut microbiota. A diet rich in vegetables, high in fiber, and low in sugar can promote the abundance of beneficial bacteria, while an HF diet can increase the abundance of pathogenic bacteria. MedDiet, Mediterranean diet. TRE, Time-restricted eating. RF, Ramadan fasting. CDHFD, Cold drink and high-fat diet. HF, High-fat. HS, High-sucrose.

Cold drinks and HF diets are associated with increased systemic inflammatory responses and mucosal barrier dysfunction [213]. Eggs are nutrient-rich, and in an aging mouse model, the abundances of *Blautia*, *Odoribacter*, and *Alistipes* significantly increased in mice fed with eggs [214]. Phenylalanine and tryptophan from diet are metabolized by *R. gnavus* to produce phenylethylamine and indole, which activate enterochromaffin cells in the gut to synthesize serotonin [215]. Studies confirm that the diversity of the gut microbiota is modulated by diet (Figure 5). HF and HS diets are an important factor in the occurrence and development of DM and its complications. A diet rich in fat and sugar can aggravate DM, leading to a decrease in *Turicibacter*, *Ileibacterium*, and *Bifidobacterium*, while increasing the abundances of *Romboutsia*, *Lactococcus*, and *Enterococcus* [216]. An HFD and high-carbohydrate diet can increase the production of SCFAs by promoting the fermentation process of gut microbiota [217]. In HF diet-fed mice, resistant starch type 1 derived from potatoes increased the abundance of *Akkermansia*, while decreasing the abundance of *Muribaculaceae* [218]. Therefore, dietary interventions that regulate microbial balance are feasible and promising strategies for disease management and treatment [219,220,221,222].

**Table 5:** The impact of dietary types on gut microbiota and its potential functions.

Type of Diet	Gut Microbiota Abundance		Function	References
	Increase	Decrease		
MedDiet	*Roseburia*, *Bacteroides*, *Faecalibacterium*, *Akkermansia*, *Bifidobacterium*, *Lachnospiraceae_UCG.001*	*Eubacterium hallii group and Dorea*, *Blautia*, *Romboutsia*, *Ruminococcus*, *Prevotella 9*	Promotes the growth of probiotics, lowers blood glucose, and has anti-inflammatory effects	[208]
Japanese diet	*Lachnospiracea*, *Gemmiger*, *Faecalibacterium*	*Alloprevotella*, *Bifidobacterium*, *Actinomyces*, *Parabacteroides*	Reduces the risk of diabetes, improves blood glucose control	[209]
TRE	*Faecalibacterium*, *Dialister*	*Alloprevotella*, *Prevotella*	Enhances insulin sensitivity, reduces body fat, and optimizes metabolism	[210]
RF	*Faecalibacterium*, *Roseburia*, *Akkermansia*, *Bacteroides*, *Allobaculum*, *Blautia*	*Prevotella 9*	Improves insulin sensitivity, promotes weight loss, and reduces inflammation	[210]
*C. morifolium*	*Akkermansia*, *Bacteroidales*, *Rikenellaceae*	*Clostridium*, *Faecalibaculum*	Reduces the risk of diabetes, improves blood glucose control	[211]
Capsaicin	*Akkermansia*, *Anaerotruncus*	*Streptococcus*, *Alistipes*, *Faecalibacterium*, *Barnesiella intestinihominis*	Improves glucose and lipid metabolism disorders	[212]
CDHFD	*/*	*Muribaculum*, *Odoribacter*	Aggravates metabolic disorders associated with diabetes	[213]
Eggs (selenium and/or zinc)	*Blautia*	*Alistipes*, *Odoribacter*	Antioxidant effects and enhances insulin sensitivity	[214]
HF and HS diet	*Romboutsia*, *Lactococcus*, *and Enterococcus*	*Turicibacter*, *Ileibacterium*, *Bifidobacterium*	Promotes insulin resistance, β-cell damage, and inflammation	[216]
HFD	*Roseburia*, *Ruminococcus gnavus*	*Bacteroides*, *Alistipes*	Promotes the proliferation of beneficial bacteria, blood glucose regulation, and anti-inflammatory effects	[217]
Vegetarian and vegan diets	*Faecalibacterium prausnitzii*	*Bacteroides fragilis*	Effective weight management, reduction of diabetes and metabolic syndrome risk	[220]
Ketogenic Diet	*Akkermansia*, *Clostridia_UCG 014*	*Bacteroides*, *Anaerostipes*, *Ruminococcus*	Effective weight management and promotion of glucose metabolism	[221]
RS	*Bifidobacterium adolescentis*, *Bifidobacterium longum*, *Ruminococcus bromii*	*Alisipes putredinis*, *Bacteroides vulgatus*, *Odoribacter* sp. *lanchnicus*	Effective weight management and promotion of glucose metabolism	[222]

MedDiet, Mediterranean diet. TRE, Time-restricted eating. RF, Ramadan fasting. *C. morifolium*, *Chrysanthemum morifolium* flower. CDHFD, Cold drink and high-fat diet. HF, High-fat. HS, High-sucrose. HFD, High-fiber diet. RS, resistant starch.

### 5.2. Supplement Probiotics to Improve DM and Its Complications

Probiotics can improve the balance of gut microbiota [223]. *Bifidobacteria*, a major component of breast milk, when supplemented in infants, results in abundant indole-3-lactic acid and indole lactic acid in the gut, both of which are linked to immune regulation [224]. *Bifidobacterium* also produces high concentrations of acetate in the gut lumen, inducing acidification [225]. Probiotic preparations containing *Bifidobacterium* and INU significantly upregulate the levels of 3-hydroxybutyric acid and glycolic acid [226]. Although clinical studies are limited to statistical analyses of the correlation between microbiota and metabolite levels, *Bacteroides* thetaiotaomicron significantly increased the proportion of polyunsaturated fatty acids in the liver of mice in a DN animal model [227]. In a mouse model of metabolic-associated fatty liver disease, *A. muciniphila* was found to regulate the metabolism of L-aspartic acid [228]. In addition, Probiotics have been demonstrated to lower fasting insulin, blood glucose, and homeostatic model assessment of IR levels in patients with GDM, while also enhancing the quantitative insulin sensitivity check index [229].

**Figure 6:**
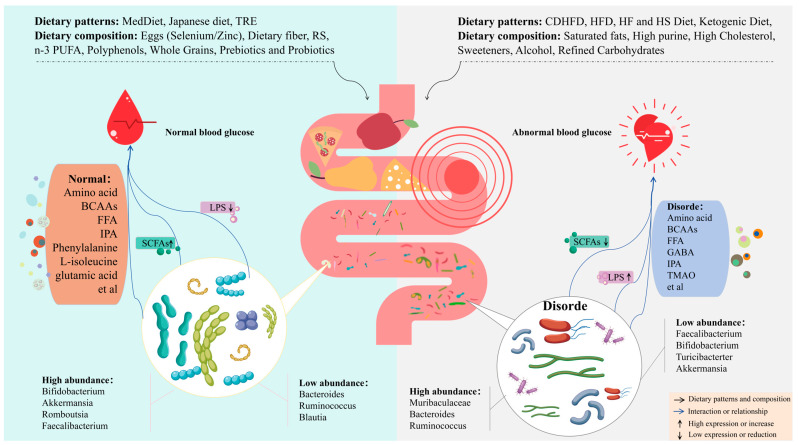
Different dietary structures affect the gut microbiota and metabolism and regulate blood glucose. In the diagram, arrows represent regulatory effects, and different background colors represent the direct impact of dietary structure on the gut microbiota. Reasonable diet on the left side and balanced gut, blood glucose homeostasis. In contrast, the right side indicates that poor dietary habits lead to gut homeostasis imbalance and opportunistic pathogens. Subsequently, under the influence of the microbiota, the body’s metabolism changes its function, thereby participating in blood glucose regulation. LPSs, Lipopolysaccharides. SCFAs, Short-chain fatty acids. BCAAs, Branched-chain amino acids. FFAs, Free fatty acids. GABA, Gamma aminobutyric acid. IPA, Indolepropionic acid. TMAO, Trimethylamine N-oxide. MedDiet, Mediterranean diet. TRE, Time-restricted eating. CDHFD, Cold drink and high-fat diet. HF, High-fat. HS, High-sucrose. HFD, High-fiber diet. RS, resistant starch. *n*-3 PUFA, Omega-3 polyunsaturated fatty acids.

Probiotics and lactic acid bacteria improve glucose metabolism disorders by increasing the expression of glucose transport proteins and regulating blood glucose and insulin levels [230]. *Lactobacillus* paracasei IMC 502 alleviates T2DM by modulating SCFAs [231]. Additionally, GPR43/41 and glucagon enhance insulin secretion by activating *GLP-1*, a process that can be initiated by probiotics [232]. Increasing levels of *Bifidobacterium*, *Lactobacillus*, and *Akkermansia* can improve glucose homeostasis. In HF diet-induced obese mice, elevated fasting blood glucose levels were observed. However, fecal transplantation of *P. copri* successfully reversed the hyperglycemia [233]. In mice fed an HF and HS diet, the abundance of *Bifidobacterium* is reduced. Nevertheless, feeding these mice *Bifidobacterium* longum 070103 fermented dairy products can reverse this condition, leading to significant decrease in fasting blood glucose and leptin levels [234]. Lactobacillus plantarum strain OLL2712 induces IL-10 production by dendritic cells derived from mice. IL-10 strongly promotes the expression of anti-inflammatory macrophages. Additionally, oral administration of OLL2712 to type 2 diabetic KKAy mice inhibits serum pro-inflammatory cytokines [235]. Therefore, the implementation of probiotics-based interventions may regulate blood glucose through intestinal microbiota, metabolites (Figure 6), insulin sensitivity, and other channels to improve DM and its complications [236].

## 6. Conclusions

This review comprehensively synthesizes evidence on T1DM, T2DM, GDM, and their complications (DR, DKD, DN), including the relationship between the gut microbiota and/or gut-microbiota-related metabolites and IR among them. This review also summarizes how TCM, dietary strategies, and probiotics target host metabolites via the gut microbiota to treat and improve DM and its complications. Future research should focus on interdisciplinary collaboration among nutritionists, microbiologists, and clinicians to develop and implement intervention strategies providing novel approaches for precision nutrition therapies for DM and related metabolic disorders.

## Figures and Tables

**Table 2:** The potential roles and regulatory signaling pathways of Key metabolites in DM and its complications.

Metabolite	Regulating Signal Pathways	Function	References
BCAAs	*Akt2*	Promoting insulin secretion and β-cell dysfunction, dual inflammatory regulation	[117,118,119,121]
	*INFGR1/JAK1/STAT1*		
	*GCN2/ATF6/PPAR-α*		
	*mTOR*		
SCFAs	*GPR 43* */NF-kappaB/MAPK*	Enhancing insulin sensitivity, β-cell function, lowering blood glucose, and anti-inflammatory effects	[124,125,126,127,128,130]
	*NLRP3/Caspase-1*		
	*STING/NF-κB/p65*		
	*GLP-1/GLP-1R/cAMP/PKA/CREB/INS*		
	*HDAC3-H3K27ac-PPAR-γ*		
IPA	*GPR109A*	Maintain intestinal homeostasis, enhance insulin secretion, and reduce insulin resistance	[132,135,136]
	AHR/NF-κB		
	SIRT1/PGC-1α		
	PXR		
TMAO	*PI3K/Akt/mTOR*	Promoting inflammation, inhibiting insulin signaling suppression, and damaging β-cells	[137,138]
	*MAPK/NF-κB*		
	*PERK-FoxO1*		
Phytosphingosine	*MAPK*	Improving metabolic disorders in diabetes and anti-inflammatory effects	[139]
	*NF-κB*		
Madecassic acid	*NF-κB*	Anti-inflammatory effects and regulating lipid metabolism	[140]
Ginsenosides	*AMPK/Akt*	Regulating hepatic glucose metabolism, alleviating inflammation and oxidative stress	[142,143,146]
	*PPAR-γ*		
	*Sirt1/PGC-1α*		
EPA	*ROS/JUN*	Increasing insulin sensitivity, promoting pancreatic β-cell function, and anti-inflammatory effects	[147,148,149]
	*miR-1a-3p/sFRP1/Wnt/PCP-JNK*		
	*AMPK*		
Chenodeoxycholic acid	*ROS/p38 MAPK/DGAT1*	Improving glucose and lipid metabolism, protecting pancreatic β-cell function	[151,152]
	*FXR-MLCK*		
Lupeol	*AMPK/NF-κB*	Antioxidant, anti-inflammatory, and protective effects on pancreatic β-cells	[153,154]
	*FXR*		

BCAAs, Branched-chain amino acids. SCFAs, Short-chain fatty acids. IPA, Indolepropionic acid. TMAO, Trimethylamine N-oxide. EPA, Eicosapentaenoic acid.

## Data Availability

No new data were created or analyzed in this study.

## Abbreviations

DM	Diabetes mellitus
T2DM	Type 2 diabetes mellitus
T1DM	Type 1 diabetes mellitus
GDM	Gestational diabetes mellitus
β-cells	Beta cells
IR	Insulin resistance
DR	Diabetic retinopathy
DKD	Diabetic kidney disease
DN	Diabetes neuropathy
DCD	Diabetic cardiovascular disease
HFD	High-fiber diet
HbA1c	Glycated hemoglobin
LPS	Lipopolysaccharides
SCFAs	Short-chain fatty acids
BCAAs	Branched-chain amino acids
IPA	Indolepropionic acid
TCM	Traditional Chinese medicine
MR	Mendelian randomization
*A. muciniphila*	*Akkermansia muciniphila*
2-HB	2-Hydroxybutyrate
VEGF	Vascular endothelial growth factor
DPN	Diabetic peripheral neuropathy
Lp299v	Lactobacillus reuteri 299v
*R. gnavus*	*Ruminococcus gnavus*
TMAO	Trimethylamine N-oxide
*B. coccoides*	*Blautia coccoides*
AGEs	Advanced glycation end-products
RAGE	Receptor for advanced glycation end-products
GPR	G protein-coupled receptors
HF	High-fat
NETs	Neutrophil extracellular traps
EPA	Eicosapentaenoic acid
NOD mice	Non-obese diabetic mice
PA	Propionic acid
BA	Butyric acid.
MedDiet	Mediterranean diet
*C. morifolium*	*Chrysanthemum morifolium* flower
HS	High-sugar
CDHFD	Cold drink and high-fat diet
TRE	Time-restricted eating
RF	Ramadan fasting
FFA	Free fatty acids
GABA	Gamma aminobutyric acid
GLP-1	Glucagon-like peptide-1
GIP	Glucose-dependent insulin-dependent polypeptide
*n*-3 PUFA	Omega-3 polyunsaturated fatty acids
